# Correction: The Correlation Between NAFLD and Serum Uric Acid to Serum Creatinine Ratio

**DOI:** 10.1371/journal.pone.0294801

**Published:** 2023-11-16

**Authors:** Jangwon Choi, Hyun Joe, Jung-Eun Oh, Yong-Jin Cho, Hwang-Sik Shin, Nam Hun Heo

The following information is missing from the Funding statement: This work was supported by the Soonchunhyang University Research Fund. HJ received this funding under the grant agreement number 2023–0067.

The following statement was incorrectly added to the Acknowledgment section: We would like to thank Editage (www.editage.co.kr) for English language editing.
