# Supplementary material for: The correlation between NAFLD and serum uric acid to serum creatinine ratio
Source: PLoS One. 2023 Jul 18;18(7):e0288666. doi: 10.1371/journal.pone.0288666 (PMC10353803; doi:10.1371/journal.pone.0288666)
Supplement: Attachment — Submitted filename: Response to Reviewers.docx [file pone.0288666.s001.docx]

We, the authors, appreciate the thoughtful and detailed reviews of the reviewers.

Normality tests were conducted on continuous variables using both the Kolmogorov-Smirnov test and the Shapiro-Wilk test. Student’s t-test, or one-way analysis of variance, was used to analyze continuous variables, whereas Pearson's chi-square test was used to analyze categorical variables. This correction was reflected in the manuscript.(page 9, 153-156)

In response to reviewer #2's comment, I have added previous findings(page 22, 295-296) and reference(37).

The authors have not received specific funding for this work so far. After this manuscript is published, we are planning to apply the research fund program of the Soonchunhyang University Industry-University Cooperation Foundation. This content was corrected in the manuscript.

The figure and table numbering has been corrected.

And all figures are revised.

All relevant data are within the manuscript.

*** No Supporting Information files.

Journal Requirements:

#1. Ensure that your manuscript meets PLOS ONE's style requirements: Confirmed

#2. financial disclosure: Confirmed

#3. Data Availability statement: All relevant data are within the manuscript.

#4. Clarify the number of Figures/Tables uploaded in your manuscript and PDF file.: Confirmed

#5. Include a caption for figure 3.: Confirmed

#6. Includ a copy of Tables 5 and 6 which you refer to in your text on page 21.: Confirmed

#7. Review your reference list to ensure that it is complete and correct.: Confirmed

I look forward to your positive reply.
